# Insights into the olaparib-mediated cell death mechanisms in canine hematological malignancies: a different fate for CLBL-1 and GL-1 cell lines

**DOI:** 10.3389/fvets.2026.1725824

**Published:** 2026-02-06

**Authors:** Greta Mucignat, Ewa Dejnaka, Marianna Pauletto, Rosa Maria Lopparelli, Mery Giantin, Aleksandra Pawlak, Mauro Dacasto

**Affiliations:** 1 Department of Comparative Biomedicine and Food Science, University of Padua -Agripolis, Legnaro, Italy; 2 Department of Pharmacology and Toxicology, Wrocław University of Environmental and Life Sciences, Wrocław, Poland; 3 Department of Physiology and Pharmacology, University of Georgia, Athens, GA, United States; 4 SMART Pharmacology, Precision One Health Initiative, University of Georgia, Athens, GA, United States

**Keywords:** apoptosis, dog, leukemia, lymphoma, olaparib, pyroptosis

## Abstract

**Introduction:**

Olaparib (OLA) is a poly ADP-ribose polymerase inhibitor (PARPi) indicated for solid cancers harboring *BRCA1/2* mutations. Recent evidences suggest that sensitivity to PARPis may also be influenced by other factors that impair the DNA repair mechanisms. Since various hematological malignancies exhibit these types of defects, this study aims to investigate further the mechanism of action of OLA in CLBL-1 and GL-1 canine cell lines, which showed different sensitivities to this PARPi.

**Methods:**

CLBL-1 and GL-1 cell lines were exposed to OLA (12.5, 25, and 50 μM) for 24 and 48 h and were subjected to preliminary cell death evaluations by flow cytometry. Then, both immunoblotting for the assessment of Bcl-2 and Bcl-XL, and RNA-seq were carried out after 24 h of exposure to OLA 25 and 50 μM. As for whole-transcriptome analysis, reads were pseudo-aligned (Kallisto) to the reference transcriptome, and differential gene expression (DGE) and functional analyses were performed with edgeR and clusterProfiler R packages.

**Results:**

The percentage of annexin V-positive cells after 24 h of incubation with OLA 50 μM was ~10%, increasing to ~40% in CLBL-1 cells and ~30% in GL-1 cells at 48 h. Bcl-2 and Bcl-XL expression increased after 24 h of incubation in CLBL-1 cells but decreased in GL-1 cells. DGE and functional analyses showed that, in CLBL-1 cells, the main processes affected by OLA were stress (e.g., *ATF3, CEBPB*) and apoptosis (e.g., *BAX, BBC3*). Conversely, in GL-1 cells, the regulation of tumor necrosis factor and interferon response-related terms, along with the upregulation of genes such as *IL6, TNF, IFIT3, GSDME*, and *IL18*, indicated the induction of pyroptosis.

**Discussion:**

The comprehensive transcriptomic analysis helped clarify the distinct mechanisms of OLA-induced cell death in CLBL-1 and GL-1 cells, which showed different sensitivities to OLA. Indeed, this PARPi appeared to interact with immune checkpoints, stress sensors, and interfere with cell proliferation, leading to various types of cell death. As canine lymphoma is a significant concern in veterinary oncology and a valuable model for its human counterpart, this study further confirms the potential of PARPi as a therapeutic approach in hematological malignancies in both species.

## 1 Introduction

The earliest molecular-targeted drugs approved for cancer treatment in human medicine were trastuzumab and imatinib in 1998 and 2001, respectively. Since then, numerous small-molecule inhibitors and monoclonal antibodies have been developed and approved for clinical use in cancer patients. In veterinary oncology, the concept of targeted therapy is more recent, with masitinib and toceranib being the first and only molecular-targeted anti-cancer drugs approved for animals (1).

Within the concept of targeted therapy, synthetic lethality has gained a significant role in human medicine. “Synthetic lethality” refers to simultaneous defects that cause cell death. The DNA damage response (DDR) is the primary pathway exploited by synthetic lethality in solid malignancies, and poly-ADP-ribose polymerases (PARPs) are among the most researched targets involved in this process. Four PARPi, namely OLA, rucaparib, niraparib, and talazoparib, have been approved by the Food and Drug Administration (FDA) in recent years for treating *BRCA1/2* mutant tumors. Indeed, cells harboring deleterious mutations in homologous recombination repair (HRR) genes are more susceptible to PARPi activity (2, 3).

Overall, BReast CAncer gene 1/2 (*BRCA1/2*) variants are less studied in veterinary medicine; nevertheless, in both human and companion animals, they have been primarily examined in solid tumors (e.g., mammary and ovarian cancers), as *BRCA* deficiency is rare in hematological malignancies (3, 4). Nonetheless, the use of PARPi has been reported in preclinical and early-phase clinical studies involving hematological cancers; additionally, germline or somatic mutations in HRR-associated genes have been identified in various hematological malignancies (5–9). Interestingly, it has also been described that the effectiveness of PARPi extends to cancers lacking *BRCA* deficiency, suggesting the involvement of other HRR genes (10). Therefore, PARPis could represent a promising therapeutic option for treating hematological cancers with HRR defects.

In this respect, the dog represents a valuable model for lymphoid tumors, which are among the most frequently diagnosed canine malignancies. Indeed, canine lymphoma represents a significant portion of canine cancers and shares many similarities with human non-Hodgkin lymphoma (11).

CLBL-1 and GL-1 are two commonly used canine lymphoid cancer cell lines, for which transcriptomic, immunophenotypic, and cytogenetic data are available (12–14). CLBL-1 cells are derived from canine diffuse large B-cell lymphoma (DLBCL) and show a B-cell immunophenotype and a monoclonal immunoglobulin heavy locus (*IGH*) gene rearrangement (15). GL-1 cells are derived from canine acute leukemia (16) and were originally classified immunophenotypically as B-cell lymphoid in origin. However, an abnormal monoclonal T cell receptor gamma locus (*TCRG*) gene rearrangement was later discovered (15). A previous study showed that CLBL-1 and GL-1 cell lines harbor DDR variants, and when compared with other two canine cancer lymphoid cell lines (CLB70 and CNK-89 cells), they were found to be the most sensitive and resistant to OLA treatment, respectively (17). Taking these first evaluations as a starting point, in the present study, the mechanism of action of OLA in these two *in vitro* models was further explored through the application of RNA-seq methodologies.

## 2 Materials and methods

### 2.1 Reagents

Roswell Park Memorial Institute (RPMI) 1640 medium was supplied by the Institute of Immunology and Experimental Therapy (Polish Academy of Sciences, Wrocław, Poland). L-glutamine, penicillin, streptomycin, RIPA Lysis buffer, and SigmaFAST Protease Inhibitor Cocktail were purchased from Sigma-Aldrich (Steinheim, Germany). Fetal bovine serum (FBS) was from Gibco (Thermo Fisher Scientific, Grand Island, NY, USA). OLA was provided by Selleckchem (Cologne, Germany) and, for flow cytometry evaluations, Annexin V (AnnV)-FITC was from Immunostep (Salamanca, Spain).

As concerns reagents for immunoblotting, Pierce™ Western Blot Signal Enhancer and Pierce™ ECL Western Blotting Substrate were from Thermo Scientific (Rockford, IL, USA). As for antibodies, anti-Bcl-XL primary antibody (54H6, 2764) was obtained from Cell Signaling Technology (Danvers, MA, USA), anti-Bcl-2 (sc-7382), and anti-β-actin clone C4 (sc-47778) antibodies from Santa Cruz Biotechnology (Santa Cruz, CA, USA). Goat anti-mouse Immunoglobulins/HRP (P0447) and Goat Anti-Rabbit Immunoglobulins/HRP (P0448) from Dako (Agilent; Santa Clara, CA, USA) were used as secondary antibodies.

TRIzol Reagent and RNeasy mini Kit used for RNA extraction were provided by Thermo Fisher Scientific (Waltham, MA, USA) and Qiagen (Hilden, Germany), respectively. For quantitative and qualitative RNA evaluation, the Qubit RNA Assay kit was purchased by Life Technologies (Carlsbad, CA, USA) and TapeStation RNA ScreenTape & Reagents by Agilent Technologies (Santa Clara, CA, USA).

### 2.2 Cell lines

The CLBL-1 cell line was obtained from Barbara C. Rütgen (Institute of Immunology, Department of Pathobiology, University of Veterinary Medicine, Vienna, Austria), while the GL-1 cells were obtained from Yasuhito Fujino and Hajime Tsujimoto (University of Tokyo, Department of Veterinary Internal Medicine). The CLBL-1 and GL-1 cell lines were maintained in RPMI 1640 medium supplemented with 2 mM L-glutamine, 100 U/ml penicillin, 100 μg/ml streptomycin, and 10% (GL-1) or 20% (CLBL-1) heat-inactivated FBS.

### 2.3 Apoptosis assessment by flow cytometry

Cells were seeded at a density of 1 × 10^5^/ml in 96-well plates (TPP, Trasadingen, Switzerland), and then incubated for 24 and 48 h with OLA 12.5, 25, and 50 μM. The cells were then collected, suspended in a binding buffer, and stained with AnnV-FITC and propidium iodide (PI, final concentration, 1 μg/ml). Flow cytometric analysis was performed immediately using a FACSCalibur flow cytometer (Becton Dickinson, Heidelberg, Germany) and analysis was done using the Cell Quest 3.1f software.

### 2.4 Cell incubation

Based on the results obtained through flow cytometry investigations, 25 and 50 μM were chosen for the following evaluations aimed to explore more deeply the cell death mechanism caused by OLA. For both immunoblotting and RNA-seq analyses, three experimental conditions for each cell line were considered: cells exposed to OLA 25 and 50 μM for 24 h (identified as GL1_T25; CLBL1_T25; GL1_T50; CLBL1_T50) and the untreated cells (identified as GL1_CTRL; CLBL1_CTRL).

### 2.5 Protein extraction and immunoblotting

A total of 5 × 10^6^ cells were rinsed with cold PBS, lysed with RIPA Lysis buffer with SigmaFAST Protease Inhibitor Cocktail, and incubated for 20 min on ice. After centrifuging at 10,000 rpm at 4 °C for 12 min, SDS sample buffer was added to clear the supernatants, and the samples were boiled at 95 °C for 5 min. Gel electrophoresis, transfer, enhancer treatment and blocking were performed as reported in Pasaol et al (17). After blocking, the membranes were incubated overnight at 4 °C with primary antibodies and then for 90 min at RT with secondary ones. Rabbit anti-Bcl (B cell lymphoma)-XL, mouse anti-Bcl-2 (dilution 1:1,000), and mouse anti-β-actin clone C4 (dilution 1:2,000) were used as primary antibodies, while goat anti-mouse immunoglobulins/HRP at 1:20,000 and goat anti-rabbit immunoglobulins/HRP at 1:10,000 as secondary ones. The reaction was developed using Pierce™ ECL Western Blotting Substrate. Membrane visualization was performed using ChemiDoc Touch Instruments (exposure: first image, 5 s; last image, 120 s; images, 5; BioRad). For protein expression quantification, normalization with a single housekeeping protein (β-actin) was performed using Image LabTM software (v.6.1.0; BioRad).

Specifically, the intensity of the Bcl-2 and Bcl-XL bands was adjusted to β-actin and expressed as *n*-fold change (arbitrary units, a.u.) compared to the integrated density value of CLBL-1 cells for baseline expression evaluation, or CTRL cells, when OLA treatment was taken into consideration. In both cases, an arbitrary value of 1 was assigned to the reference condition.

The statistical analysis of the abovementioned comparisons was performed with GraphPad Prism v.10 (GraphPad Software, San Diego, CA, USA).

### 2.6 RNA-seq

#### 2.6.1 RNA extraction, library preparation, and sequencing

Samples were first lysed and homogenized using 1 ml of TRIzol Reagent. Then they were incubated for 5 min to allow complete dissociation of the nucleoprotein complex. Chloroform (200 μl) was added to each sample, which was then shaken vigorously for 1 min. After a 2 min incubation, samples were centrifuged for 20 min at 13,000 rpm at 4 °C. The upper aqueous phase was then mixed with cooled (−20 °C) 70% ethanol and transferred to a RNeasy spin column. The following part of the protocol followed the manufacturer's instructions for the RNeasy mini Kit. Total RNA was quantified by using the NanoDrop 1000 Spectrophotometer (Thermo Fisher Scientific, Waltham, MA, USA) and the Qubit RNA Assay kit, in a Qubit 2.0 Fluorometer (Life Technologies). All samples had an RNA Integrity Number (RIN) value > 7, evaluated with TapeStation (Agilent Technologies, Santa Clara, CA, USA).

Library preparation and sequencing were performed by Novogene Biotechnology (Cambridge, UK). A total of 21 tagged RNA-seq libraries were prepared and sequenced using a 150 bp stranded-specific paired-end strategy in an Illumina Novaseq 6000.

#### 2.6.2 Differential gene expression and functional analyses

For RNA-seq analysis, three biological replicates were used for CLBL-1 cells, and four biological replicates for GL-1 cells.

Raw reads underwent quality control with FastQC *s*oftware (v.0.11.9) (18) and the amount of ribosomal RNA was checked using Bowtie2 (v.2.2.9) (19) using an index built on SILVA databases of ribosomal small and large subunits for Bacteria, Archaea and Eukaryotes (https://www.arbsilva.de/no_cache/download/archive/current/Exports/). Then, low-quality reads and adapters were removed using Trimmomatic (v.0.36) (20).

Trimmed reads were pseudoaligned to the reference canine transcriptome (ROS_Cfam_1.0, Ensembl release 109) using Kallisto (v.0.48.0) (21). Transcripts were then imported in RStudio (R v.4.2.1) and collapsed to genes using the tximport package (v.1.24.0) (22) and annotations retrieved from Ensembl with the R interface biomaRt (v.2.54.0) (23).

The following steps of DGE analysis were performed using the edgeR package (v.3.38.4) (24). The data were organized in two different datasets, one for each cell line, and processed with the same approach. First, genes with very low expression level were removed (*filterByExpr*), and the remaining ones were normalized using the *calcNormFactors* function according to the trimmed mean of *M*-values (TMM).

After common and tagwise dispersion estimation (*estimateDisp*) and negative binomial generalized linear models are fitted (*glmQLFit*), differentially expressed genes (DEGs) were identified using quasi-likelihood *F*-test (*glmQLFTest*) (25), setting these contrasts: GL1_T50 vs. GL1_CTRL; GL1_T25 vs. GL1_CTRL; GL1_T50 vs. GL1_T25; CLBL1_T50 vs. CLBL1_CTRL; CLBL1_T25 vs. CLBL1_CTRL; CLBL1_T50 vs. CLBL1_T25. Looking at the preliminary results obtained for CLBL-1 cells (data not shown), we also considered a batch effect as a covariate. For the principal component analysis (PCA), the *removeBatchEffect* limma function was used for this cell line.

DEGs were defined as those with Benjamini Hochberg adjusted *p*-value (BH*p*) < 0.05 and log_2_ fold change (lfc) ≥0.59 or ≤ -0.59 from each dataset. They were graphically represented through volcano plots (EnhancedVolcano, v.1.14) (26).

DEGs were then submitted to over-representation analysis using Gene Ontology (GO)—Biological Process and Kyoto Encyclopedia of Genes and Genomes (KEGG) databases using *enrichGO* and *enrichKEGG* functions of the clusterProfiler package (v.4.4.4) (27).

## 3 Results

### 3.1 Cell death analysis

**Figure 1:**
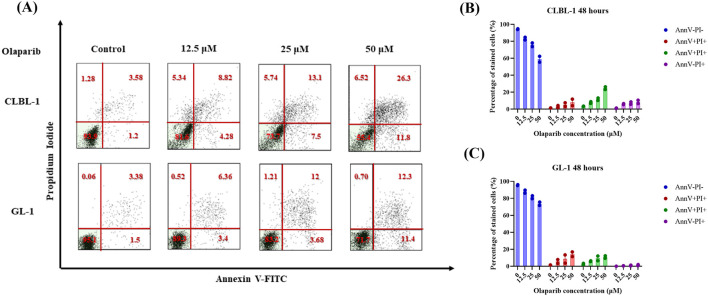
Cell death analysis of CLBL-1 and GL-1 cells treated with increasing concentrations of OLA at 48 h. Results of AnnV/PI staining after the treatment of CLBL-1 and GL-1 cells with OLA (12.5, 25, and 50 μM) for 48 h. Representative dot plots **(A)** are reported alongside histograms **(B, C)** for each cell line and condition tested. The histograms represent mean ± standard error of the mean of two independent cell culture experiments.

According to the AnnV/PI assay, OLA induced apoptosis in both CLBL-1 and GL-1 cell lines in a time- and concentration-dependent manner. The percentage of AnnV-positive cells after 24 h of incubation was small, oscillating around 10% for each cell line. After 48 h of incubation, the induction of apoptosis was much stronger, and differences in the sensitivity of individual cell lines were visible. At the highest concentration tested (50 μM), OLA induced apoptosis in approximately 40% of the CLBL-1 cells and less than 30% of the GL-1 cells. Detailed results showing the positivity to AnnV/PI staining in the tested cells, as well as the representative dot plots, are shown in Supplementary Figure 1 and Figure 1, for the 24 and 48 h of incubation, respectively.

### 3.2 Immunoblotting

Comparing the baseline expression of Bcl-2 and Bcl-XL in the two cell lines, GL-1 cells were found to have higher levels of both proteins than CLBL-1 cells. Specifically, after normalizing band intensity to actin and setting the integrated mean density of CLBL-1 cells to an arbitrary value of 1, GL-1 cells expressed 2.3 and 7.2 higher levels of Bcl-2 and Bcl-XL, respectively, compared to CLBL-1 cells. This difference was statistically significant only for Bcl-2 (*p* < 0.05; Supplementary Figure 2).

**Figure 2:**
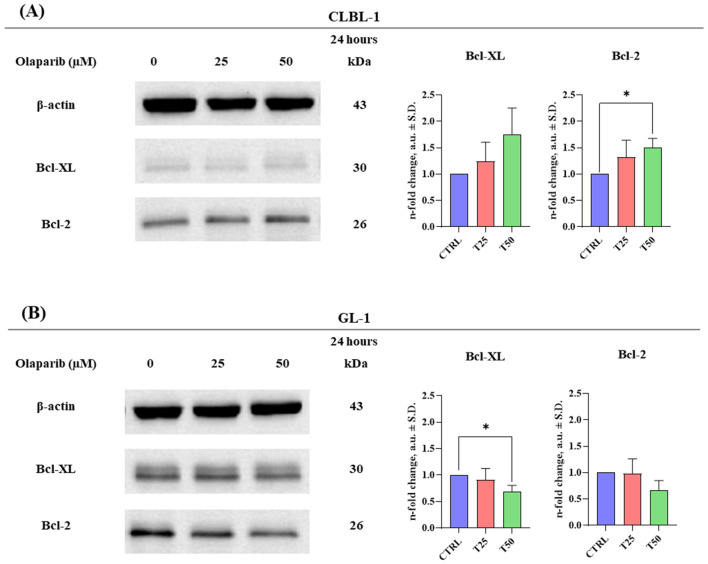
The effect of OLA treatment on Bcl-2 and Bcl-XL protein expression in CLBL-1 and GL-1 cell lines. **(A)** Bcl-2 and Bcl-XL protein expression in CLBL-1 cells after 24 h of incubation with OLA 25 and 50 μM. **(B)** Bcl-2 and Bcl-XL protein expression in GL-1 cells after 24 h of incubation with OLA 25 and 50 μM. Quantification was performed by normalizing the expression level of the protein of interest to the expression level of the loading control, β-actin. Mean and standard deviations were calculated based on three independent experiments. The statistical significance (*p < 0.05) is calculated using a one sample *t*-test.

Furthermore, at 24 h, OLA 25 and 50 μM caused an increase in Bcl-XL and Bcl-2 protein expression in the CLBL-1 cell line; the result was statistically significant (*p* < 0.05) only for Bcl-2 with the highest concentration of OLA tested (Figure 2A). Conversely, in the GL-1 cell line, a progressive decrease of both proteins was detected after OLA treatment. The effect was statistically significant (*p* < 0.05) only with OLA 50 μM for Bcl-XL (Figure 2B).

### 3.3 Differential gene expression and functional analyses

A mean of 25,936,908 reads/sample were processed, with 19,009,498 (73%) pseudo-aligned to the reference transcriptome. Pre-processing and mapping results were reported in Supplementary Table 1 for both CLBL-1 and GL-1 cell lines.

#### 3.3.1 Differential gene expression and functional analyses of the CLBL-1 cell line

**Figure 3:**
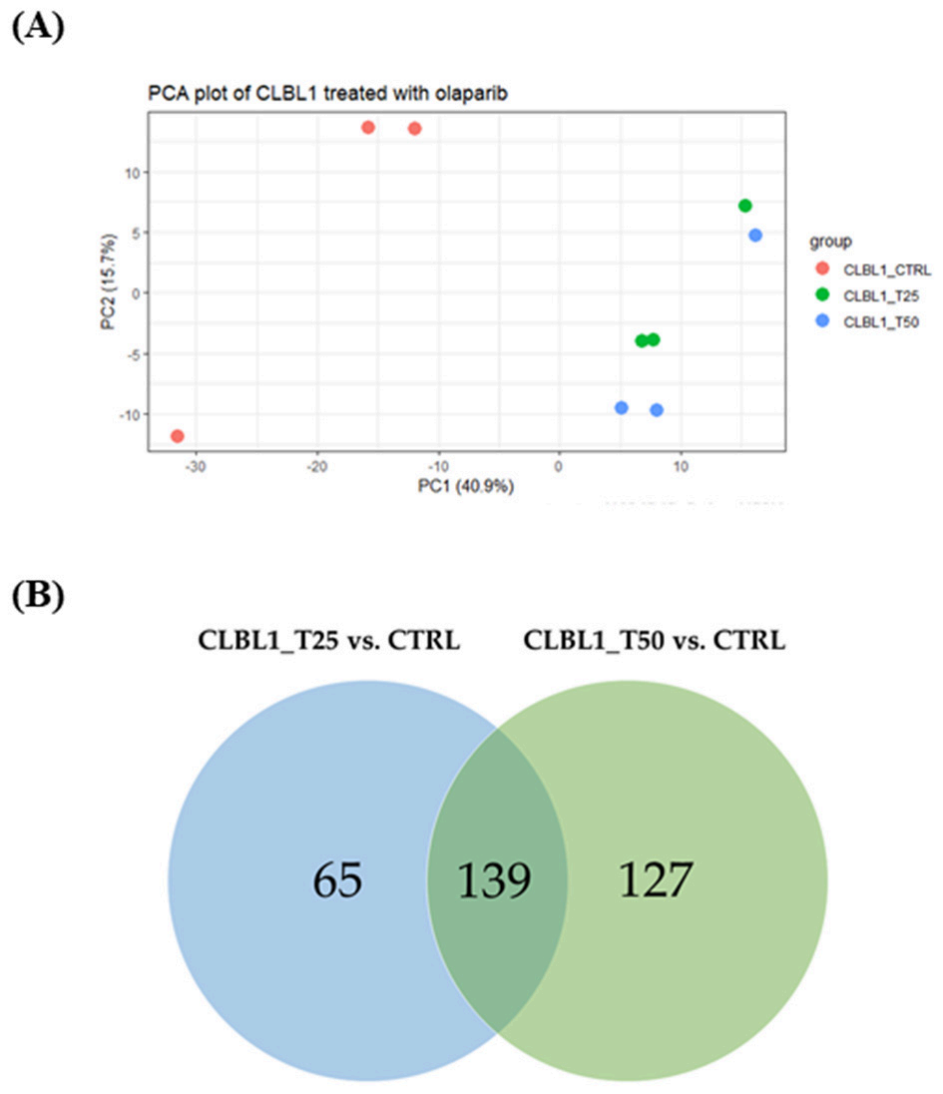
PCA plot and Venn diagram of CLBL-1 cells treated with OLA. **(A)** PCA plot of the dataset including CLBL1_CTRL, CLBL1_T25, and CLBL1_T50 experimental groups. **(B)** Venn diagram with the number of common DEGs between CLBL1_T25 vs. CTRL and CLBL1_T50 vs. CTRL.

CLBL-1 cells, after batch effect correction, clustered according to the experimental conditions, as shown in the PCA (see Figure 3A). According to the differential gene expression analysis, 204 and 266 DEGs were identified considering CLBL1_T25 vs. CTRL and CLBL1_T50 vs. CTRL contrasts, respectively (Table 1). Among DEGs, 139 genes were found in common, as shown by the Venn diagram (Figure 3B). No significant differences were observed between the two OLA concentrations (Table 1).

**Table 1:** Number of DEGs for CLBL1_T25 vs. CTRL, CLBL1_T50 vs. CTRL, and CLBL1_T50 vs. CLBL1_T25 comparisons.

**DEGs category**	**CLBL1_T25 vs. CTRL**	**CLBL1_T50 vs. CTRL**	**CLBL1_T50 vs. CLBL1_T25**
Total DEGs	204	266	0
Upregulated DEGs	178	219	0
Downregulated DEGs	26	47	0
Common DEGs	139		

The table reports the significant DEGs obtained from the statistical analysis (BHp < 0.05 and |lfc| > 0.59) of the following contrasts: CLBL1_T25 vs. CTRL, CLBL1_T50 vs. CTRL, and CLBL1_T50 vs. CLBL1_T25.

The lists of DEGs obtained from CLBL1_T25 vs. CTRL and CLBL1_T50 vs. CTRL comparisons (Supplementary Table 2) were used to perform the GO and KEGG overrepresentation analysis. The complete output of the functional analysis is reported in Supplementary Table 3.

**Figure 4:**
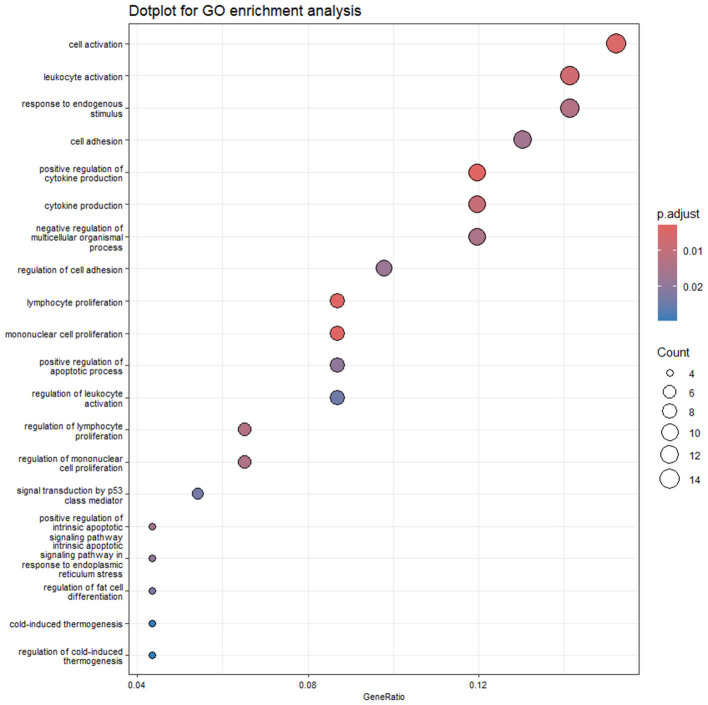
Dot plot of GO enrichment of CLBL1_T50 vs. CTRL comparison. The plot reports the 20 most significant GO terms enriched by the DEGs resulting from CLBL1_T50 vs. CTRL comparison. The color gradient is related to the level of significance, adjusted with the Benjamini–Hochberg method.

As concerns GO enrichment analysis, 3 (Supplementary Figure 3) and 25 terms were enriched by DEGs that were modulated by OLA 25 and 50 μM, respectively. As expected, considering the high number of DEGs shared between the two comparisons, the terms enriched by OLA at the lowest concentration were recalling those obtained for the highest concentration. The 20 most significant GO terms enriched by the highest concentration are reported in Figure 4.

**Figure 5:**
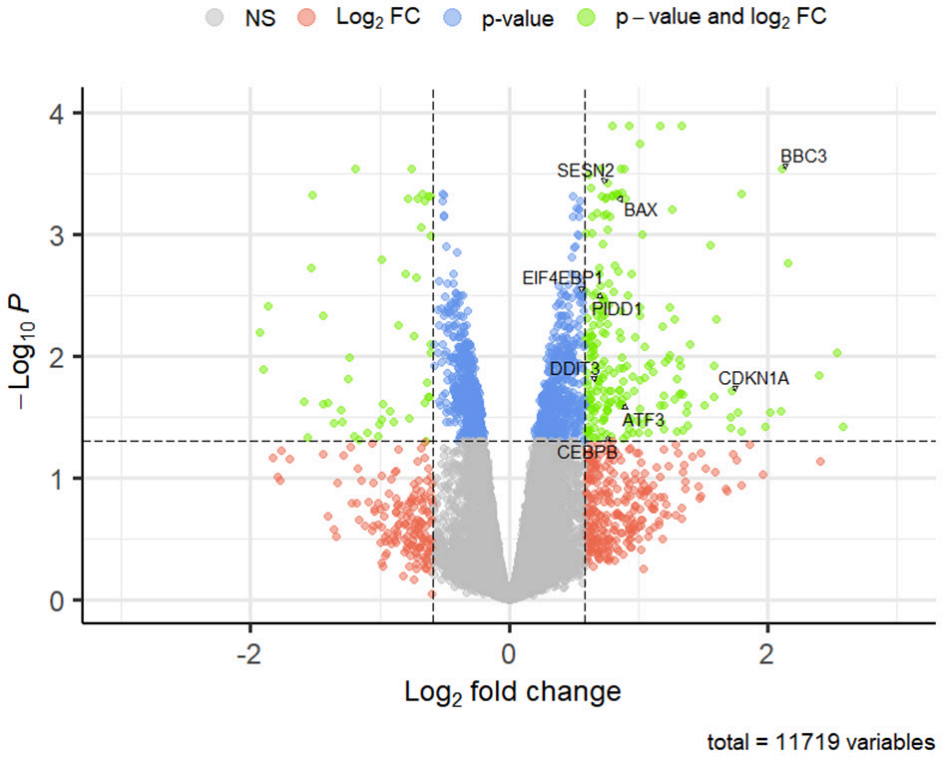
Volcano plot of DEGs in CLBL1_T50 vs. CTRL comparison. Lfc are reported on the x axis; logarithms (base 10) of BHp are reported on the y axis; dashed lines set the threshold of significance (BHp ≤ 0.05), and log_2_ fold-change (lfc ≥ 0.59 or ≤ −0.59). Significantly regulated genes are shown as green dots in the upper left and upper right quadrants (down and upregulated genes, respectively). Genes that do not meet lfc, BHp cut-off or both are represented by blue, orange and gray dots, respectively.

Among the GO terms referring to the CLBL1_T50 vs. CTRL comparison, it's interesting to consider those related to cytokine production (GO:0001819; GO:0001816), leukocyte activation (GO:0001775; GO:0045321; GO:0002694), signal transduction by p53 class mediator (GO:0072331), apoptotic process (GO:2001244; GO:0070059; GO:0043065), and endoplasmic reticulum stress (GO:0070059; GO:0034976). Among genes enriching these pathways, Bcl-2 Binding Component 3 (*BBC3*), Bcl-2 Associated X, Apoptosis Regulator (*BAX*), DNA Damage-Inducible Transcript 3 (*DDIT3*), Sestrin 2 (*SESN2*), CCAAT Enhancer Binding Protein Beta (*CEBPB*), and Cyclin-Dependent Kinase Inhibitor 1A (*CDKN1A*) are worth mentioning. Moreover, although not enriching a specific term, but possibly associated with the previously mentioned processes, p53-Induced Death Domain Protein 1 (*PIDD1*), Activating Transcription Factor 3 (*ATF3*), and Eukaryotic Translation Initiation Factor 4E Binding Protein 1 (*EIF4EBP1*) were also differentially regulated in CLBL1_T50 vs. CTRL comparison. The DEGs mentioned above are labeled in the volcano plot in Figure 5.

As for KEGG overrepresentation, 5 (Supplementary Figure 4) and 4 (Supplementary Figure 5) KEGG pathways were found to be modulated by OLA at the lowest and highest concentrations, respectively. KEGG pathways were quite overlapping between the two comparisons, and were mostly related to cancer and immune-related subcategories, e.g., cytokine-cytokine receptor interaction (cfa04060), neutrophil extracellular trap formation (cfa04613), and viral carcinogenesis (cfa05203).

#### 3.3.2 Differential gene expression and functional analyses of the GL-1 cell line

**Figure 6:**
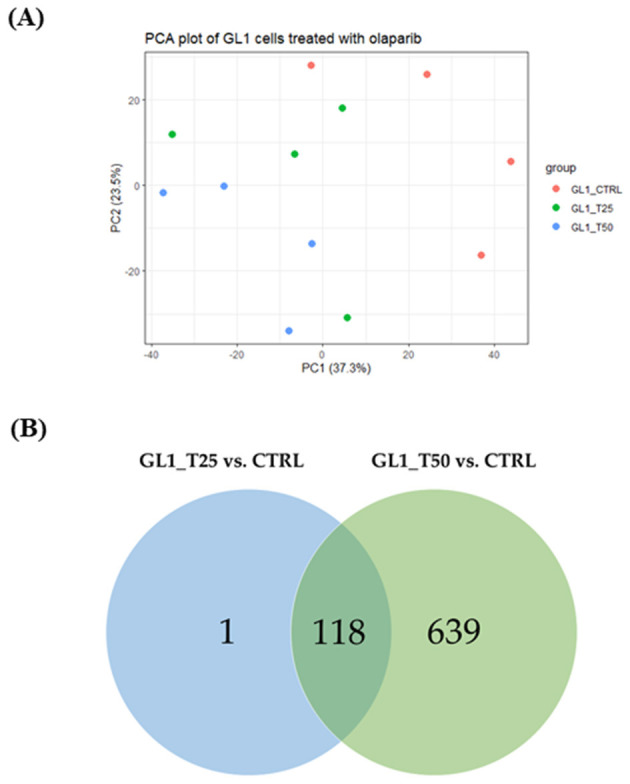
PCA plot and Venn diagram of GL-1 cells treated with OLA. **(A)** PCA plot of the dataset including GL1_CTRL, GL1_T25, and GL1_T50 experimental groups. **(B)** Venn diagram with the number of common DEGs between GL1_T25 vs. CTRL and GL1_T50 vs. CTRL.

The PCA of the GL-1 cell line data (Figure 6A) shows how samples clustered according to the experimental conditions. From the DGE analysis, 757 and 119 DEGs were obtained considering GL1_T50 vs. CTRL and GL1_T25 vs. CTRL contrasts, respectively (Table 2). No significant difference was observed between the two OLA concentrations (Table 2). The comparison of the two lists of DEGs (GL1_T50 vs. CTRL and GL1_T25 vs. CTRL) showed that almost all the genes modulated by OLA 25 μM were also modulated by OLA 50 μM, suggesting a dose-dependent effect (see the Venn diagram in Figure 6B).

**Table 2:** Number of DEGs for GL1_T25 vs. CTRL, GL1_T50 vs. CTRL, and GL-1_T50 vs. GL-1_T25 comparisons.

**DEGs category**	**GL1_T25 vs. CTRL**	**GL1_T50 vs. CTRL**	**GL1_T50 vs. GL1_T25**
Total DEGs	119	757	0
Upregulated DEGs	113	654	0
Downregulated DEGs	6	103	0
Common DEGs	118		

The table reports the significant DEGs obtained from the statistical analysis (BHp < 0.05 and |lfc| > 0.59) of the following contrasts: GL1_T25 vs. CTRL, GL1_T50 vs. CTRL, and GL1_T50 vs. GL1_T25.

**Figure 7:**
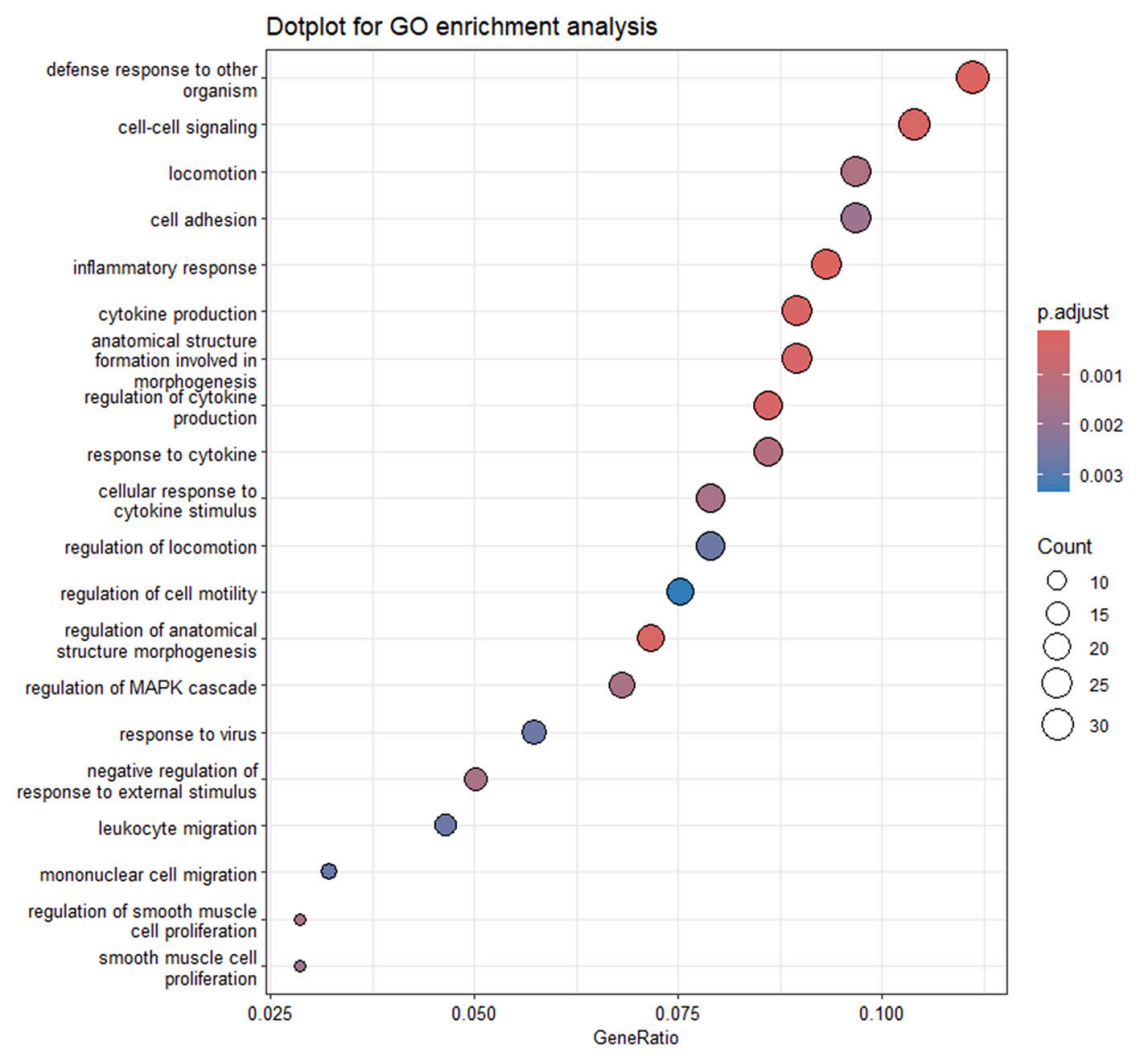
Dot plot of GO enrichment of GL1_T50 vs. CTRL comparison. The plot reports the 20 most significant GO terms enriched by the DEGs resulting from GL1_T50 vs. CTRL comparison. The color gradient is related to the level of significance, adjusted with the Benjamini–Hochberg method.

The list of DEGs of GL1_T25 vs. CTRL and GL1_T50 vs. CTRL was used to perform the GO and KEGG overrepresentation analysis. The complete output of the functional analysis is reported in Supplementary Table 5. A total of 61 and 37 GO terms were enriched with OLA at the highest and lowest concentration, respectively. As expected, given the high percentage of DEGs shared between the two comparisons, the results of the functional analysis were quite similar between the lowest and the highest doses, as can be seen by comparing the 20 most significant enriched terms (Supplementary Figure 6 and Figure 7).

**Figure 8:**
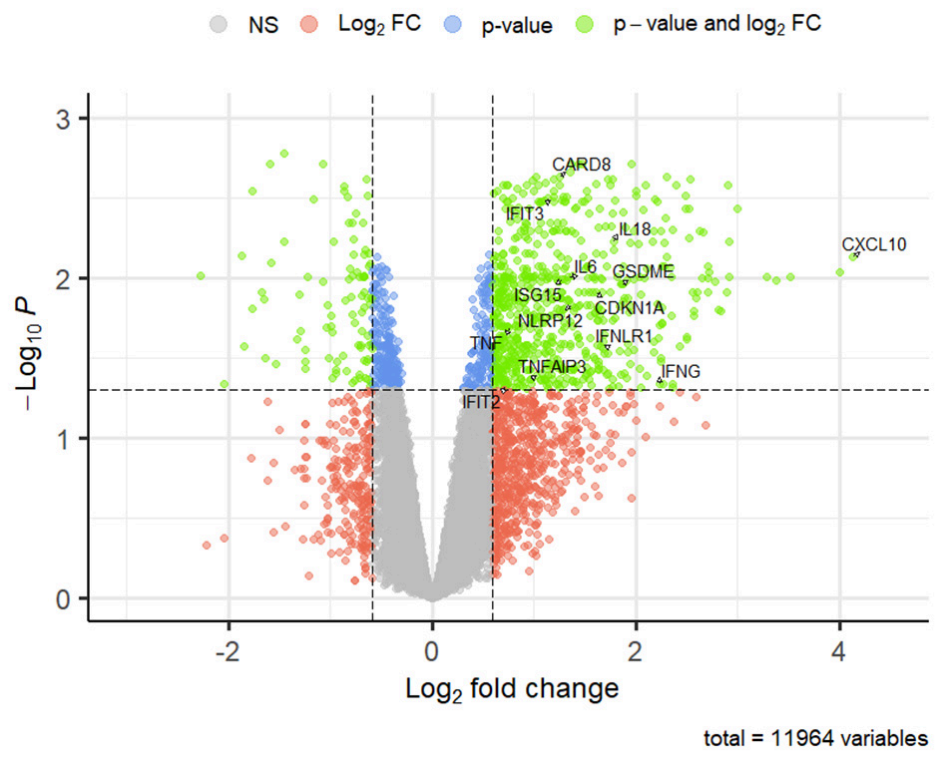
Volcano plot of DEGs in GL1_T50 vs. CTRL comparison. Lfc are reported on the x axis; logarithms (base 10) of BHp are reported on the y axis; dashed lines set the threshold of significance (BHp ≤ 0.05), and log_2_ fold-change (lfc ≥ 0.59 or ≤ −0.59). Significantly regulated genes are shown as green dots in the upper left and upper right quadrants (down and upregulated genes, respectively). Genes that do not meet lfc, BHp cut-off, or both are represented by blue, orange, and gray dots, respectively.

For the sake of completeness, the full results for both concentrations are provided in Supplementary Table 5; nevertheless, hereby we will only refer to the results for the OLA highest concentration. Terms related to the inflammatory (GO:0006954), immune response (GO:0001816, GO:0001817, GO:0034097), locomotion (GO:0040011, GO:0040012), apoptosis (GO:1904019, GO:1904035), and angiogenesis (GO:0016525, GO:1901343, GO:20001819, GO:0001568) were observed. Among genes enriching these terms, C-X-C Motif Chemokine Ligand 10 (*CXCL10*), Interferon Gamma (*IFNG*), Interferon-Stimulated Gene 15 (*ISG15*), NLR Family Pyrin Domain Containing 12 (*NLPR12*), Tumor Necrosis Factor (*TNF*), TNF Alpha Induced Protein 3 (*TNFAIP3*), Interleukins 6 (*IL6*), 18 (*IL18*), and Gasdermin E (*GSDME*) are worth of mention. Furthermore, looking closer to the list of DEGs modulated by this OLA concentration, it is worth mentioning also the upregulation of *CDKN1A*, Interferon Induced Protein with Tetratricopeptide Repeats 2 (*IFIT2)*, and 3 (*IFIT3)*, Interferon Lambda Receptor 1 (*IFNLR1*), and Caspase Recruitment Domain Family Member 8 (*CARD8*). The DEGs mentioned above are labeled in the volcano plot in Figure 8.

As concerns KEGG enrichment, 0 and 21 pathways were enriched by DEGs resulting from GL1_T25 vs. CTRL and GL1_T50 vs. CTRL comparisons, respectively (Supplementary Figure 7). Pathways mostly belonging to signaling molecules and interaction, immune system, and disease subcategories were enriched, such as hematopoietic cell lineage (cfa04640), viral protein interaction with cytokine and cytokine receptor (cfa04061), cytokine-cytokine receptor interaction (cfa04060), systemic lupus erythematosus (cfa05322), and allograft rejection (cfa05330).

## 4 Discussion

The DDR pathway is frequently dysregulated in lymphomas and leukemias, opening up the possibility to exploit this vulnerability as a target for the treatment of these malignancies (7, 28, 29). PARP1 is a crucial regulator of the DDR pathway, particularly in base excision repair (BER), where it facilitates the recruitment of other DDR proteins. Inhibiting PARP catalytic activity with PARPis impairs repair of single-strand breaks (SSBs), leading to replication fork collapse and double-strand breaks (DSBs). PARPis also trap PARPs on DNA to varying extents depending on the compound, and modulate the immune response by activating interferon (IFN) gene pathways. Among FDA-approved PARPis, OLA is the most extensively studied in hematological cancers (7, 8). It targets multiple PARP isoforms (PARP1, PARP2, PARP3, PARP4), inhibiting their catalytic activity and demonstrating a higher trapping efficiency than rucaparib and veliparib, but lower than niraparib and talazoparib (7).

The two cell lines used in this study, namely CLBL-1 and GL-1, were chosen because they exhibited different responses to OLA. Indeed, our previous publication showed that OLA 25 and 50 μM caused DNA damage in both cell lines but differentially affected the metabolic activity and proliferation rates of the two cell models (17). Specifically, GL-1 cells showed lower sensitivity to this PARPi compared to CLBL-1 cells (17). GL-1 cells are generally more resistant to various DNA-damaging agents, such as etoposide, doxorubicin, and berzosertib (30, 31). Conversely, they show higher sensitivity to alkylating agents, such as chlorambucil, cyclophosphamide, and cisplatin (30–32). One reason for the resistance might be P-glycoprotein (PgP) expression, which can explain why some substrates of this transporter (etoposide, doxorubicin, OLA, and berzosertib) are less effective in GL-1 cells (33–35). To confirm or refute this hypothesis, we looked at *ABCB1* mRNA expression in the RNA-seq datasets of both cell lines, and we verified that either GL-1 or CLBL-1 cells showed a low mRNA expression profile of this transporter. This evidence agrees with previous studies showing that in both CLBL-1 and GL-1 cell lines, the *ABCB1* promoter region was found to be hypermethylated, and the treatment with hypomethylating drugs increased *ABCB1* mRNA expression (36). Therefore, we may speculate that in GL-1 cells, the highest resistance to OLA is not associated with PgP expression but is probably attributable to other factors.

Among potential alternative causes, single-nucleotide variants (SNVs) and copy number alterations in genes involved in the DDR pathway were previously described (13, 17). Among them, a heterozygous loss of phosphatase and tensin homolog (*PTEN)* resulting in a significant reduction of *PTEN* mRNA expression was observed in CLBL-1 cells (13). This evidence is consistent with our own findings, showing that GL-1 cells express higher levels of *PTEN* than CLBL-1 (Supplementary Figure 8).

Other possible causes of OLA resistance in the GL-1 cell line may be inferred from the human counterpart (human leukemias) and from the recent advances in treating these malignancies with PARPis. Fms-related receptor tyrosine kinase 3 (*FLT3*) is, in fact, one of the most frequently mutated genes in human myeloid leukemias; an internal tandem duplication (*FLT3*-ITD) associated with resistance to PARPis has been recently identified (7, 37, 38). Interestingly, the GL-1 cell line harbors multiple copies of *FLT3* and shows loss of the wild-type *FLT3* sequence, consistent with amplification of the *FLT3*-ITD allele (14, 39), in accordance with our RNA-seq results showing a higher *FLT3* mRNA expression in GL-1 cells compared to CLBL-1 (Supplementary Figure 8).

Another genetic variant that confers resistance to PARPis in human leukemia cells is the *KMT2A*-*MLLT3* (lysine methyltransferase 2A-MLLT3 super elongation complex subunit) gene fusion (7, 38). Interestingly, GL-1 cells showed a higher expression profile of some *KMT2A*-*MLLT3* targets, i.e., *HOXA9, HOXA10*, and *MEIS1*, compared to CLBL-1 cells (Supplementary Figure 8), suggesting the possible presence of this rearrangement in the GL-1 cell line.

Overall, the rearrangements, copy number alterations, and SNVs mentioned above could trigger the activation or inhibition of distinct molecular mechanisms, which may, at least in part, explain the varying sensitivities. Therefore, this study conducted RNA-seq analyses to gain a clearer understanding of the differential molecular responses occurring in GL-1 and CLBL-1 cells after OLA treatment.

The preliminary evaluation of the apoptotic markers coupled with RNA-seq investigations revealed interesting complementary results to the information previously obtained (17). The AnnV/PI analysis showed that in CLBL-1 cells, the fraction of apoptotic cells increased over time, reaching a value of 40% at 48 h of incubation. Unexpectedly, the expression of Bcl-2 and Bcl-XL increased in the first 24 h of treatment, suggesting a post-transcriptional or a post-translational mechanism of regulation because of the lack of modulation at the mRNA level. Bcl-2 and Bcl-XL are well-known anti-apoptotic proteins, which are upregulated following longer periods of OLA exposure (40, 41). Higher Bcl-XL expression is reported in chemotherapy-resistant cells, supported by the synergistic effect of Bcl-2 family inhibitors with other drugs, PARPis included (40–43). This is in line with the higher baseline expression of Bcl-2/XL detected in the more resistant GL-1 cell line, both at mRNA (Supplementary Figure 8) and protein levels. On the other hand, the increase in Bcl-2/XL expression in CLBL-1 cells after OLA treatment, considering the short period of exposure to the PARPi, could be interpreted as an initial attempt to counterbalance the apoptotic signals triggered by the treatment. Indeed, at 24 h, the transcription of the pro-apoptotic *BAX* and *BBC3* (PUMA) was induced, together with the cell cycle regulator *CDKN1A* (p21), leading us to infer the conserved activity of p53. The activation of the p53 pathway could also have caused the upregulation of *PIDD1, SESN2, ATF3, CEBPB, DDIT3* (CHOP), and *EIF4BP1*. Despite the well-known and prevalent DNA-damage activity of OLA, an ATF4-mediated integrated stress response (ISR) cannot be excluded *a priori*. In fact, ATF4, one of the main transcriptional effectors of ISR, can modulate the expression of the abovementioned *SESN2, ATF3, CEBPB, DDIT3*, and *EIF4EBP1* transcripts; moreover, its function is interconnected with p53 as it restores p53 transcriptomic targets in p53-mutant cells (44, 45).

In GL-1 cells, the mechanism appears different; none of the genes mentioned above were significantly modulated by the treatment, except for *CDKN1A*, which was upregulated by OLA. The non-significant regulation of the canonical p53 targets, e.g., *BAX* and *BBC3*, could be a hint of a partial loss-of-function (LOF) of p53 (46), which retains transcriptional activity only on some responding elements, like the ones in *CDKN1A*, as in our case. Indeed, in GL-1 cells, p53 shows a homozygous mutation in the DNA-binding domain, specifically on Arg237 [p.(Arg237Trp): (17)], that corresponds to codon Arg249 of human p53 (see the protein sequence alignment in Supplementary Figure 9). In humans, this site is a hotspot for p53 mutations, and specifically p.Arg249Ser is the most frequent substitution, responsible for a LOF (47). Thus, the mutation p.(Arg237Trp) may explain why in GL-1 cells the metabolic activity and the proliferation are less influenced by the treatment (17), and why the fraction of AnnV-positive cells here observed is more contained. However, to confirm the role of p53 in the resistance mechanism to OLA in GL-1 cells, validation studies, such as restoring the functional p53 in GL-1 cells or knocking it out in CLBL-1 cells, will be necessary.

For the GL-1 cell line, RNA-seq data adds additional insights to the phenotypic results. Apart from a possible LOF of p53, transcriptomic analyses revealed a strong inflammatory response associated with the upregulation of genes related to IFN and TNF, such as *IFIT3, IFIT2, IFNG, IFNLR1, ISG15, TNF*, and *IL6*. In addition, the simultaneous upregulation of *GSDME, IL18, CARD8*, and *NLRP12* suggested that another mechanism of cell death was taking place. Pyroptosis is a proinflammatory form of cell death involving membrane pore formation, mediated by gasdermins (GSDMs), which cause the release of inflammatory cytokines like IL-1β and IL-18. Both TNF and INF-γ were found to participate in this process. The induction of *IFIT3*, along with *IFIT1*, and the inhibition of Bcl-2 were found to be crucial for activating GSDME in human leukemia and myeloma cells (48). On the other hand, TNF could promote pyroptosis by activating caspase (CASP)8, which can directly activate GSDMD or indirectly activate GSDME through CASP3 (49). Indeed, GSDME is known to be specifically activated by CASP3 in both mice and humans (50), and this may also apply to canine GSDME, which contains the CASP3 cleavage motif (51). This pore-forming protein is often silenced in cancer tissues (52) and can serve as a switch between apoptosis and pyroptosis depending on its expression level (52). Thus, we hypothesize that GL-1 cells may have the potential to switch from apoptosis to pyroptosis due to the higher basal *GSDME* expression compared to CLBL-1 cells (Supplementary Figure 8). Additionally, we speculate that dual inhibition of Bcl-2/XL triggered GSDM-mediated cell death, as previously reported (53). To reach a definitive conclusion, GSDMs cleavage, CASPs activation, and IL-18 release should be assessed at the protein level, along with the use of specific GSDMs inhibitors or gene knockout experiments. The induction of CASP-dependent pyroptosis by PARPis has already been reported in human ovarian, breast cancer, and *BRCA1*-mutated cervical carcinoma cell lines (49, 54). However, the limited cross-reactivity of commercial antibodies against veterinary species may hinder the replication of validation assays from human studies.

Besides this, in GL-1 cells, pyroptosis appeared transcriptionally activated by OLA at 24 h, without showing phenotypic effects for the subsequent 24 h (i.e., low proportion of AnnV-positive cells for 48 h). This may suggest the need to increase OLA concentrations or exposure time to observe potential delayed effects. Furthermore, since PARP1-trapping plays a pivotal role in GSDME cleavage (54), the use of other PARPi with higher PARP-trapping activity than OLA (talazoparib and niraparib) might potentiate this mechanism of cell death and thereby deepen understanding of the phenomenon.

Regardless of these future perspectives, the GL-1 cell line remains resistant to OLA treatment. In fact, OLA alone does not appear to be effective in GL-1 cells (17), indicating an intrinsic chemoresistance that may extend beyond this specific PARPi. Specifically, the previously reported *FLT3*-ITD (39), the suspected *KMT2A*-*MLLT3* gene fusion, or the SNV in *TP53* (17), could explain the resistance mechanism of the GL-1 cell line, though further assays are needed to confirm their presence and role. The observed resistance to apoptosis, along with the potential involvement of alternative cell death pathways such as pyroptosis, highlights the need to explore different therapeutic strategies. Understanding the dominant mode of cell death in GL-1 cells could guide the selection of more effective treatments for leukemia. For example, PARPis with stronger PARP-trapping capacity, or combination therapies, such as the co-treatment with doxorubicin, DNA methyltransferase (DNMT), Bcl-2, and FLT3-inhibitors, or immune modulators (7, 17, 37, 41, 55) may enhance the therapeutic response in hematological malignancies.

## 5 Conclusion

PARPis have shown the potential to be a promising therapeutic strategy for lymphoma and leukemia in both human and canine patients. However, a comprehensive characterization of the genetic background is imperative for personalizing therapy and enhancing the efficacy of these molecules in treating hematological malignancies.

In the present study, two canine tumor cell lines, CLBL-1 and GL-1, derived from DLBCL and B-cell leukemia, exhibited different sensitivities to OLA, as indicated by AnnV/PI positivity. This difference may be attributable to rearrangements, copy-number alterations, and SNVs previously reported in these cell lines (13, 17, 39). Moreover, the evaluation of anti-apoptotic markers, e.g., Bcl-2 and Bcl-XL, and RNA-seq analysis disclosed further aspects related to different mechanisms of action and cell death. While in the CLBL-1 cell line apoptosis was induced by OLA, in the less sensitive B-cell leukemia model, pyroptosis appeared transcriptionally modulated, suggesting also potential strategies to enhance the efficacy of these molecules.

Despite the interesting insights obtained in two canine cell models mirroring the human counterpart, this study has some limitations, as it is based primarily on cell death and resistance mechanisms inferred from transcriptomic analyses. Validation of the proposed mechanisms across a larger number of cell lines and xenograft models will be required to confirm the present findings and strengthen their translatability.

## Data Availability

Raw Illumina sequencing data have been deposited in GenBank (SRA) under the BioProject accession PRJNA1242827.
