# Supplementary material for: Insights into the olaparib-mediated cell death mechanisms in canine hematological malignancies: a different fate for CLBL-1 and GL-1 cell lines
Source: Front Vet Sci. 2026 Feb 6;13:1725824. doi: 10.3389/fvets.2026.1725824 (PMC12920183; doi:10.3389/fvets.2026.1725824)
Supplement: Supplementary file 1 [file Table_1.docx]

Supplementary Material

**Insights into the olaparib-mediated cell death mechanisms in canine hematological malignancies: a different fate for CLBL-1 and GL-1 cell lines**

Mucignat Greta^1†^, Dejnaka Ewa^2†^, Pauletto Marianna^1^, Lopparelli Rosa Maria^1^, Giantin Mery^1*^, Pawlak Aleksandra^2,3,4*^, Dacasto Mauro^1^

^1^Department of Comparative Biomedicine and Food Science, University of Padua, Agripolis Legnaro, Padua, Italy

^2^Department of Pharmacology and Toxicology, Faculty of Veterinary Medicine, Wrocław University of Environmental and Life Sciences, 50-375 Wrocław, Poland

^3^Department of Physiology and Pharmacology, University of Georgia, Athens, GA, USA

^4^SMART Pharmacology, Precision One Health Initiative, University of Georgia, Athens, GA, USA

^†^These authors have contributed equally to this work

*** Correspondence:**Giantin Mery

mery.giantin@unipd.it

Pawlak Aleksandra

aleksandra.pawlak@upwr.edu.pl

**Supplementary Table 1. Sequencing and mapping results of CLBL-1 and GL-1 samples.**

| Samples | Raw reads | Trimmed | % Trimmed | Pseudoaligned | % Pseudoaligned |
| --- | --- | --- | --- | --- | --- |
| CLBL1_CTRL_1 | 25,935,999 | 25,702,148 | 0.90 | 18,802,924 | 72.50 |
| CLBL1_CTRL_2 | 23,858,206 | 23,585,806 | 1.14 | 17,892,260 | 74.99 |
| CLBL1_CTRL_4 | 25,440,771 | 25,221,059 | 0.86 | 18,222,805 | 71.63 |
| CLBL1_T25_1 | 25,559,770 | 25,267,800 | 1.14 | 19,312,851 | 75.56 |
| CLBL1_T25_2 | 21,459,405 | 21,213,520 | 1.15 | 16,022,921 | 74.67 |
| CLBL1_T25_4 | 23,564,614 | 23,382,078 | 0.77 | 16,700,503 | 70.87 |
| CLBL1_T50_1 | 27,560,325 | 27,232,962 | 1.19 | 20,758,715 | 75.32 |
| CLBL1_T50_2 | 28,906,129 | 28,592,695 | 1.08 | 21,685,280 | 75.02 |
| CLBL1_T50_4 | 26,975,358 | 26,691,487 | 1.05 | 18,824,707 | 69.78 |
| GL1_CTRL_1 | 23,977,369 | 23,735,760 | 1.01 | 17,748,340 | 74.02 |
| GL1_CTRL_2 | 28,113,904 | 27,910,335 | 0.72 | 21,210,827 | 75.45 |
| GL1_CTRL_3 | 24,749,625 | 24,562,143 | 0.76 | 17,951,163 | 72.53 |
| GL1_CTRL_4 | 25,614,938 | 25,351,147 | 1.03 | 17,532,233 | 68.45 |
| GL1_T25_1 | 26,748,187 | 26,445,568 | 1.13 | 20,034,624 | 74.90 |
| GL1_T25_2 | 28,653,054 | 28,416,254 | 0.83 | 21,879,863 | 76.36 |
| GL1_T25_3 | 27,864,034 | 27,625,892 | 0.85 | 19,942,060 | 71.57 |
| GL1_T25_4 | 27,413,093 | 27,192,579 | 0.80 | 20,183,224 | 73.63 |
| GL1_T50_1 | 23,769,937 | 23,561,684 | 0.88 | 17,048,737 | 71.72 |
| GL1_T50_2 | 23,644,591 | 23,400,403 | 1.03 | 17,913,910 | 75.76 |
| GL1_T50_3 | 19,791,853 | 19,640,084 | 0.77 | 13,833,713 | 69.90 |
| GL1_T50_4 | 35,073,902 | 34,796,151 | 0.79 | 25,697,805 | 73.27 |
| Mean | 25,936,953 | 25,691,270 | 1 | 19,019,827 | 73 |

**
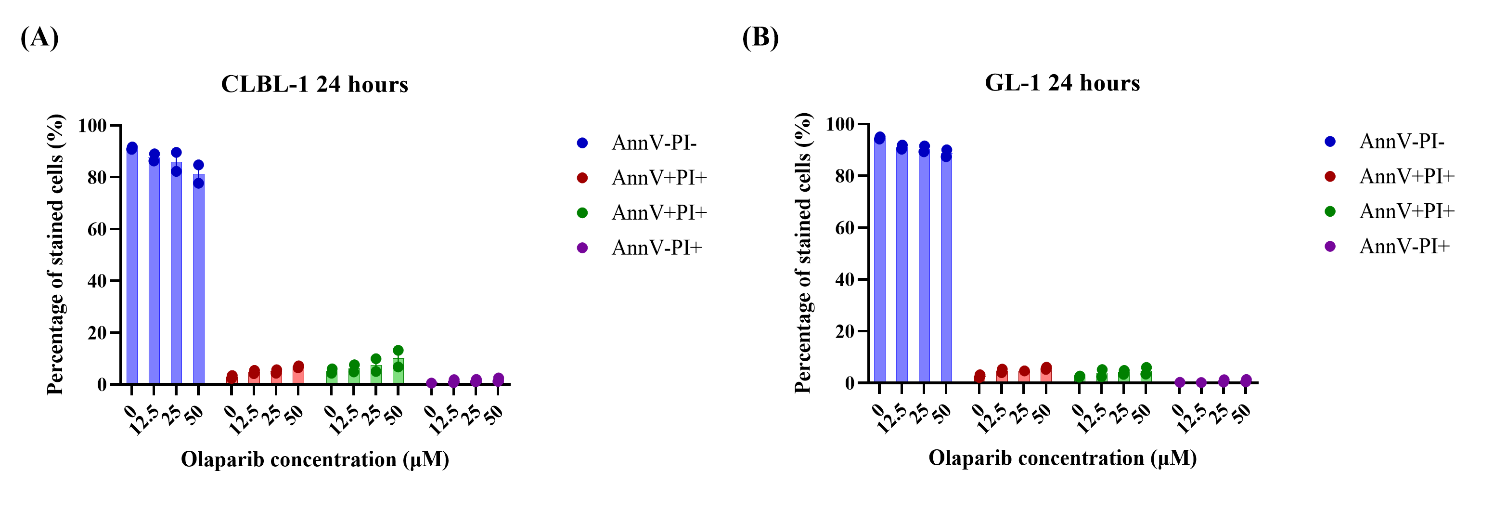
**

**Supplementary Figure 1. Cell death analysis of CLBL-1 and GL-1 cells treated with increasing concentrations of OLA at 24 hours.** Results of AnnV/PI staining after treatment for 24 hours with OLA for the CLBL-1 (A) and GL-1 (B) cell lines. The histograms represent mean ± standard error of the mean of two independent cell culture experiments.


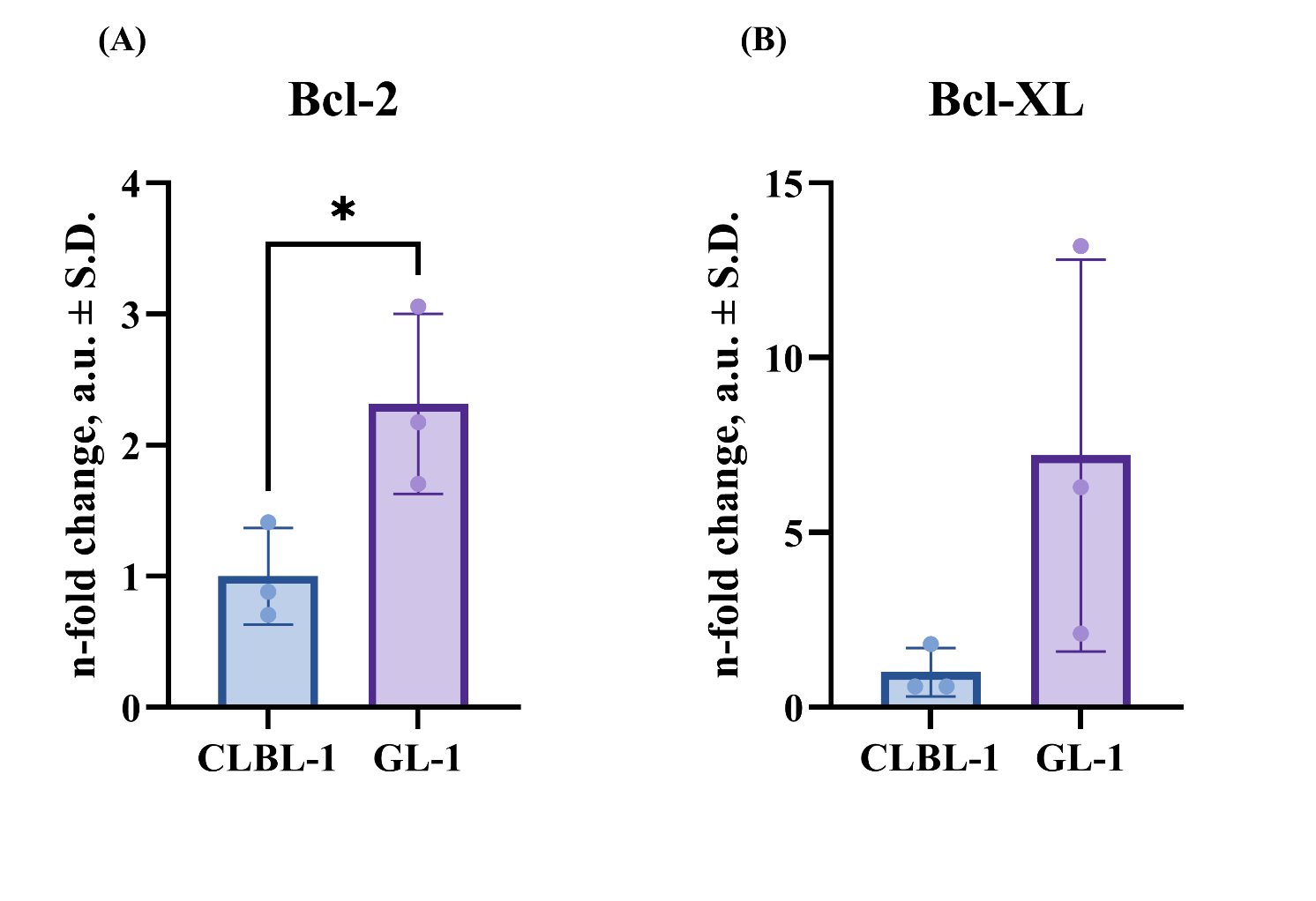


**Supplementary Figure 2. Baseline expression of Bcl-2/XL proteins in CLBL-1 and GL-1 cell lines.** Integrated density data of target Bcl-2 (A) and Bcl-XL (B) proteins, normalized on β-actin bands, are expressed as n-fold change (arbitrary units, a.u.) compared to the integrated density mean value of CLBL-1 cells to which an arbitrary value of 1 was assigned. Mean and standard deviations (S.D.) were calculated based on three independent experiments. Statistical analysis: unpaired t-test (*: *p* < 0.05)

**
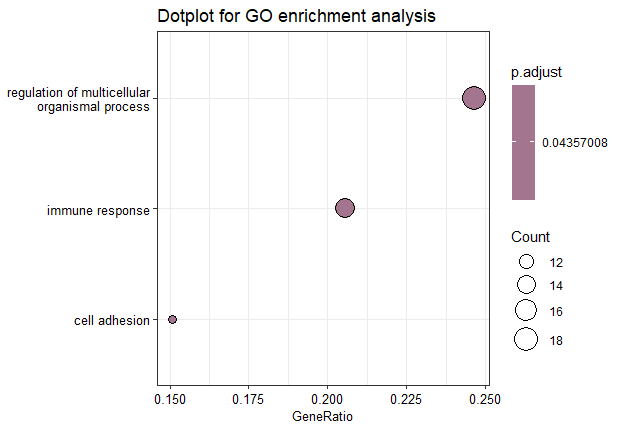
**

**Supplementary Figure 3. Dot plot of GO enrichment of CLBL1_T25 vs. CTRL comparison.** The plot reports the 20 most significant GO terms enriched by the DEGs resulting from CLBL1_T25 vs. CTRL comparison. The color gradient is related to the level of significance, adjusted with the Benjamini–Hochberg method.

**
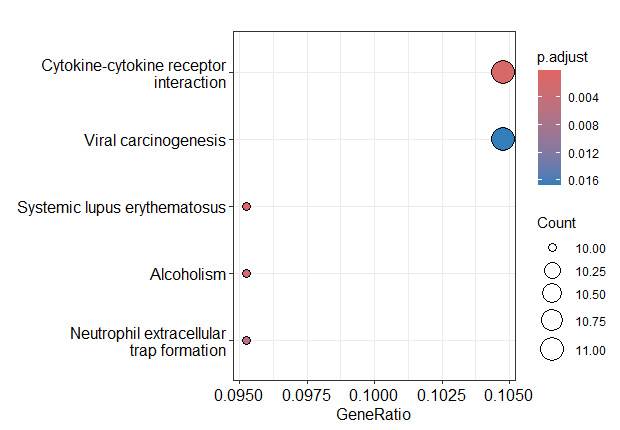
**

**Supplementary Figure 4. Dot plot of KEGG enrichment of CLBL1_T25 vs. CTRL comparison.** The plot reports the KEGG pathways enriched by the DEGs resulting from CLBL1_T25 vs. CTRL comparison. The color gradient is related to the level of significance, adjusted with the Benjamini–Hochberg method.


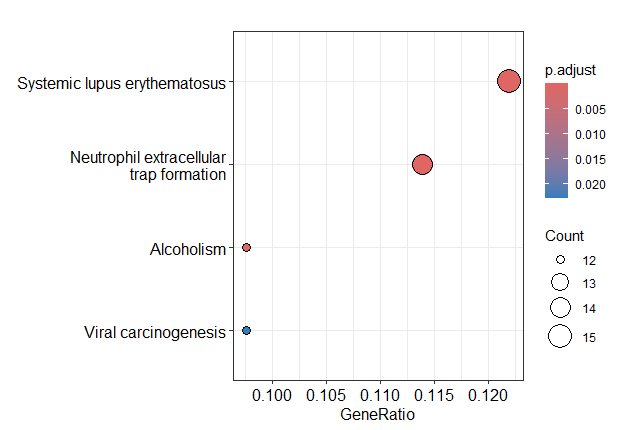


**Supplementary Figure 5. Dot plot of KEGG enrichment of CLBL1_T50 vs. CTRL comparison.** The plot reports the 4 pathways enriched by the DEGs resulting from CLBL1_T50 vs. CTRL comparison. The color gradient is related to the level of significance, adjusted with the Benjamini–Hochberg method.


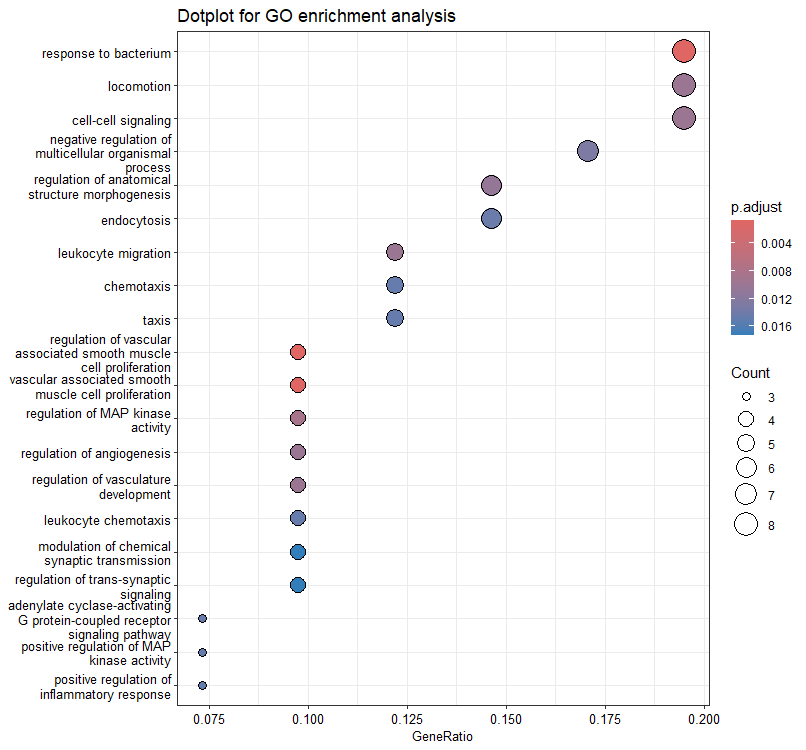


**Supplementary Figure 6. Dot plot of GO enrichment of GL1_T25 vs. CTRL comparison.** The plot reports the 20 most significant GO terms enriched by the DEGs resulting from GL1_T25 vs. CTRL comparison. The color gradient is related to the level of significance, adjusted with the Benjamini–Hochberg method.


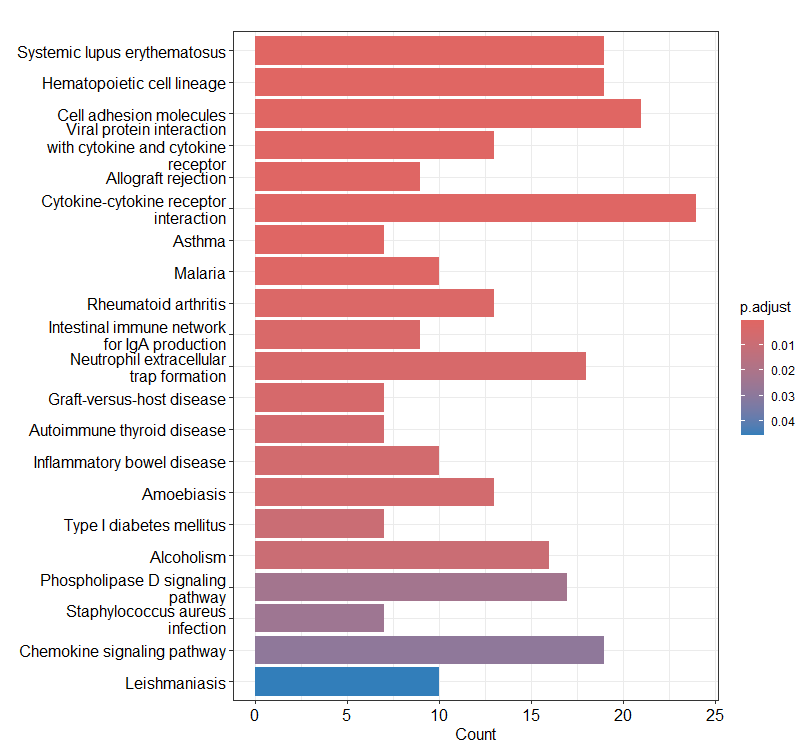


**Supplementary Figure 7. Bar plot of KEGG enrichment of GL1_T50 vs. CTRL comparison.** The plot reports all the KEGG pathways enriched by the DEGs resulting from GL1_T50 vs. CTRL comparison. The color gradient is related to the level of significance, adjusted with the Benjamini–Hochberg method.


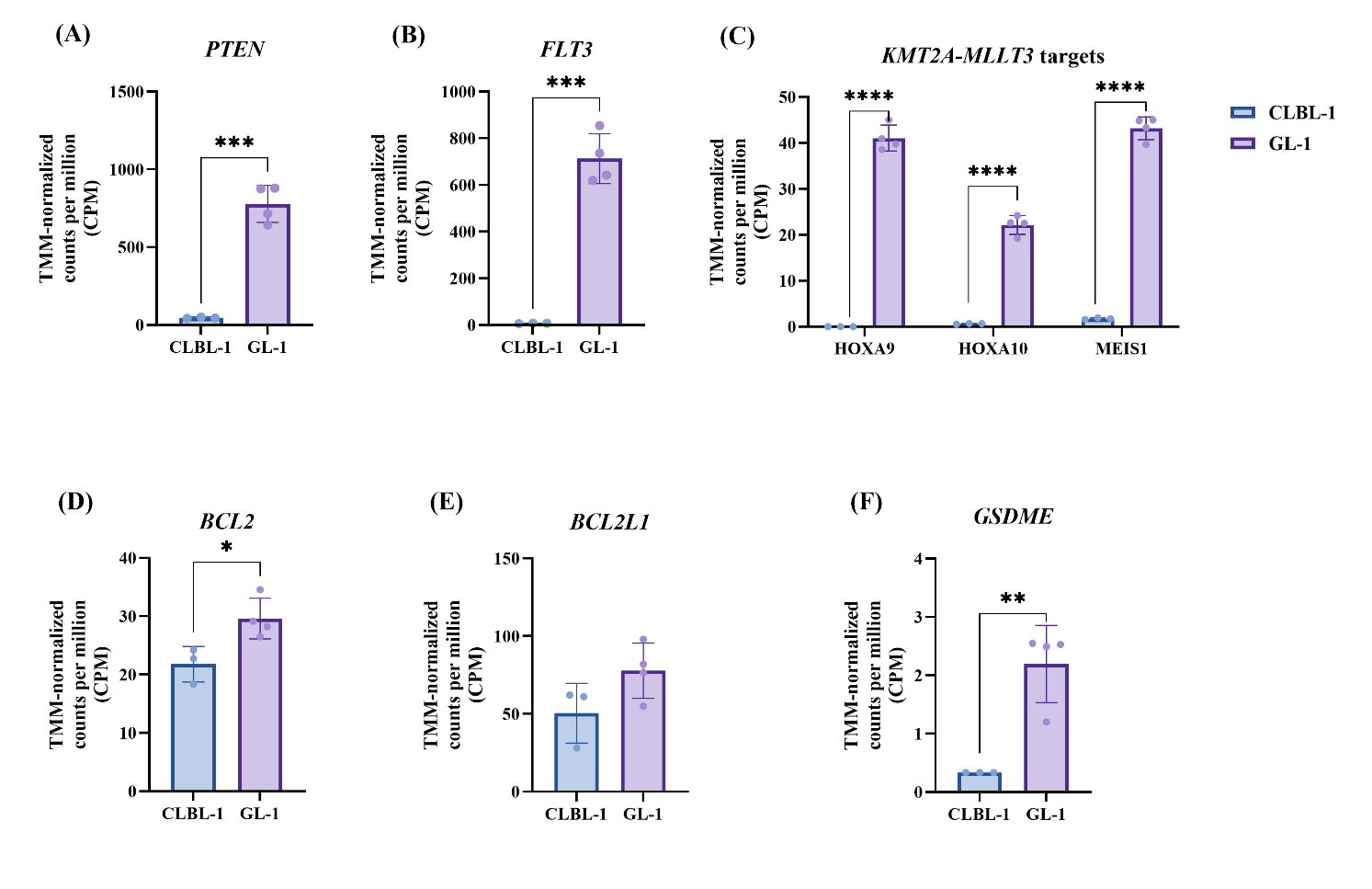


**Supplementary Figure 8. Baseline expression of *PTEN*, *FLT3*, *HOXA9*, *HOXA10*, *MEIS1*, *BCL2*, *BCL2L1,* and *GSDME* in CLBL-1 and GL-1 cell lines.** GL1_CTRL and CLBL1_CTRL samples were processed as a single dataset. Kallisto outputs were imported using tximport, low-expressed genes were filtered out, and TMM-normalization was applied. TMM-normalized counts per million (CPM) of selected genes, *PTEN* (A), *FLT3* (B), *HOXA9*, *HOXA10*, *MEIS1* (C), *BCL2* (D), *BCL2L1* (E) and *GSDME* (F), were then analyzed using unpaired t-test in Prism v.10 (GraphPad Software, San Diego, CA, USA). *: *p* < 0.05; **: *p* < 0.01. Mean and standard deviations were calculated based on three and four independent experiments for CLBL-1 and GL-1 cells, respectively.


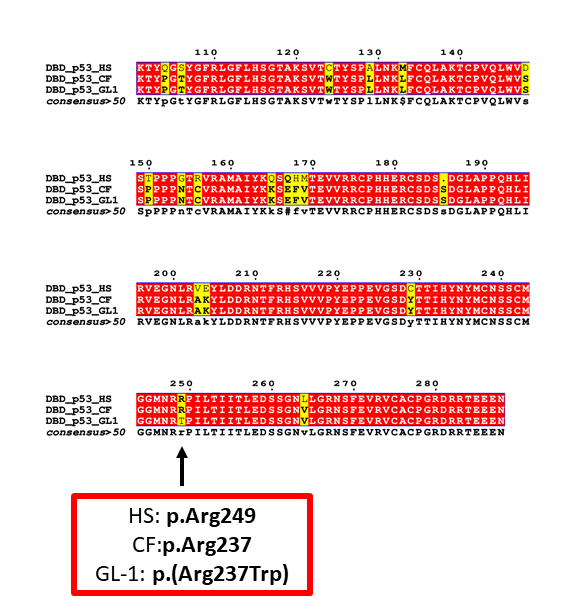


**Supplementary Figure 9. Alignment of the DNA-binding domain (DBD) of human (HS), canine (CF), and GL-1 p53.** The figure shows the alignment of the DBD (Pfam: PF00870) of p53, comparing the reference sequence (Ensembl) for human (ENSP00000269305.4) and canine (ENSCAFP00000024579.4) p53 with the predicted sequence of the GL-1 cell line. The figure was prepared by importing MultAlin output in ESPript 3.0.
